# Additive antiangiogenesis effect of ginsenoside Rg3 with low-dose metronomic temozolomide on rat glioma cells both in vivo and in vitro

**DOI:** 10.1186/s13046-015-0274-y

**Published:** 2016-02-13

**Authors:** Caixing Sun, Yang Yu, Lizhen Wang, Bin Wu, Liang Xia, Fang Feng, Zhiqiang Ling, Shihua Wang

**Affiliations:** Department of Neurosurgery, Zhejiang Cancer Hospital, Hangzhou, Zhejiang 310022 China; Zhejiang Cancer Research Institute, Zhejiang Cancer Hospital, 38 Guangji Road, Hangzhou, Zhejiang 310022 China; Department of Cancer Biology, Wake Forest School of Medicine, Medical Center Boulevard, NC, 27157 USA

**Keywords:** TMZ, RG3, Glioblastoma, Metronomic, Angiogenesis, VEGF, Allograft, rCBV

## Abstract

**Background:**

Glioblastoma is the most common and deadly primary brain tumor in adults. Low-dose,metronomic (LDM) temozolomide (TMZ) displays improved efficacy in the treatment of glioblastoma by targeting angiogenesis, but has a limited effect on recurrence. The antiangiogenesis drug ginsenoside Rg3 (RG3) is the main active ingredient of ginseng, a popular herbal medicine.

**Methods:**

Using an in vitro and a rat model of an orthotopic glioma allograft, this study was to determine whether RG3 enhanced the antiangiogenesis activity of LDM TMZ in the treatment of glioblastoma.

**Results:**

Our results showed that combined use of TMZ with RG3 displayed additive inhibition on proliferation of both human umbilical vein endothelial cells (HUVEC) and rat C6 glioma cells in vitro. They additively arrested cell cycle, increased apoptosis, and decreased VEGF-A and BCL-2 expression in HUVEC. Antiangiogenesis effect was also evaluated in the rat model of orthotopic glioma allograft, based upon markers including relative cerebral blood volume (rCBV) by magnetic resonance imaging (MRI), VEGF levels and microvessel density (MVD)/CD34 staining. LDM TMZ alone was potent in suppressing angiogenesis and tumor growth, whereas RG3 alone only had modest antiangiogenesis effects. Combined treatment significantly and additively suppressed angiogenesis, without additive inhibitory effects on allografted tumor growth.

**Conclusions:**

These data provide evidence showing the efficacy of LDM TMZ on glioma treatment. The combined additive antiangiogenesis effect suggests that RG3 has the potential to further increase the efficacy of LDM TMZ in the treatment of glioblastoma.

**Electronic supplementary material:**

The online version of this article (doi:10.1186/s13046-015-0274-y) contains supplementary material, which is available to authorized users.

## Background

Glioblastoma is the most common and lethal primary brain cancer in adults. The current standard treatment for this newly diagnosed disease is initial maximal feasible surgery with subsequent radiation plus chemotherapy [1, 2]. Temozolomide (TMZ) is the second generation of alkylating agents with excellent oral bioavailability and good penetration across the blood brain barrier [3, 4]. TMZ combined with radiotherapy improves glioblastoma patient survival rates and prolongs the progression-free survival compared with radiation therapy alone [1]. It is currently the preferred first line chemotherapeutic drug in combination with radiation in the treatment for glioblastoma. However, the five-year survival rate after the diagnosis is still less than 8 % under current standard therapy [5–7].

Glioblastoma is a highly vascularized tumor, rarely having extracranial metastases. As high as 40–50 % of glioblastoma’s have high levels of VEGF, the strongest proangiogenic factor in the induction of tumor angiogenesis. High VEGF correlates with a poor overall clinical prognosis. Glioblastoma is therefore an ideal disease for antiangiogenesis treatment [8].

The low-dose metronomic (LDM) chemotherapy is given by continuously administering a drug at a relatively low dose, with minimal intervals. LDM use of traditional drugs in cancer therapy was initially conducted in animal models with neuroblastoma [9] and Lewis lung cancer [10]. LDM use of the chemotherapeutic drug vinblastine significantly reduced the size of xenografted neuroblastoma’s and inhibited tumor angiogenesis. Metronomic vinblastine in combination with the VEGF receptor-2 antibody (DC101) led to complete regression of the established neuroblastoma and no relapse in over a 6 month period, with little toxicity [9]. In another study, the LDM use of cyclophosphamide was three fold more effective in reducing both xenografted Lewis lung carcinoma and EMT-6 breast cancer in nude mice, compared with the conventional regimen [10]. Low dose metronomic (LDM) use of the traditional chemotherapeutic drugs have been shown to target both proliferating tumor cells and endothelial cells, and minimize toxicity [11, 12].

The LDM TMZ demonstrated an antiangiogenic effect in an orthotopic glioma model [13]. Brock et al. demonstrated that a continuous daily administration of TMZ at a dose of 75 mg/m^2^/day for periods up to 6–7 weeks can be well tolerated without a significant increase in toxicity [14]. LDM TMZ has been shown to prolong glioblastoma patient progression-free interval survival, with less toxicity [15–17]. A recent meta-analysis shows that LDM TMZ improves the overall clinical benefit rate, and lengthens progression-free survival; however, no overall survival is observed between metronomic and standard regimen [17]. Therefore, it is of exceptional clinical significance to identify antiangiogenetic drugs which can further improve the efficacy of LDM TMZ to extend glioblastoma patients’ survival [18].

Ginseng is a popular herbal medicine in Asian countries used to improve health and treat certain chronic diseases [19]. Many studies have reported that ginseng promotes a wide range of pharmacologic activities in the immune, cardiovascular, endocrine, and central nervous systems. In recent years, ginseng has begun to gain significant popularity in Western societies. As the main active ingredient of ginseng, ginsenoside Rg3 (RG3) is the protopanaxadiol type of dammarane ginsenoside consisting of characteristic genuine aglycone moieties [20]. RG3 has multiple functions in immune regulation, anti-oxidation, anti-inflammation, anti-aging and anti-fatigue. Its antiangiogenesis along with anti-tumorigenic effects have been shown in several cancers [21–23]. More strikingly, RG3 has a synergistic effect with several chemotherapy drugs to reverse multi-drug resistance of tumor cells [24–26]. Its tumor suppressor effect was also observed in glioma in vitro [27, 28]. The objective of this study was to determine whether RG3 in combination with LDM TMZ is capable of enhancing antiangiogenesis activity.

## Methods

### Cell lines and reagents

Primary human umbilical vein endothelial cells (HUVECs) were obtained from ScienceCell (Beijing, China). HUVECs were maintained in endothelial cell medium (ECM) supplemented with 10 % fetal bovine serum, 4 ng/ml FGF4, 2 ng/ml VEGF165, 100 unit/ml penicillin and 100 μg/ml streptomycin. Rat C6 glioma cell line was purchased from Shanghai Institutes for Biological Sciences (Shanghai, China). Rat C6 glioma cells were maintained in RPMI 1640 medium supplemented with 10 % newborn calf serum, 100 unit/ml penicillin and 100 μg/ml streptomycin at 37 °C in a 5 % CO_2_ incubator. CellTiter 96® Queous Non-Radioactive Cell Proliferation Kit (MTS) was provided by Promega (Madison, WI). Both VEGF and CD34 antibodies were purchased from ABCAM (Hangzhou, China). The horseradish peroxidase conjugated goat anti-rabbit IgG second antibody (H + L) was purchased from MultiSciences Biotech Co., Ltd (Hangzhou, China). Primers for GAPDH, Bcl-2 and VEGF were synthesized by Invitrogen (Grand Island, NY). The primer sequences and the expected sizes were: GAPDH primer forward, CAA CTC CCT CAA GAT TGT CAG CAA and reverse, GGC ATG GAC TGT GGT CAT GA, 118 bp; Bcl-2 primer forward, CCA GCG TAT ATC GGA ATG TGG and reverse, CCA TGT GAT ACC TGC TGA GAA G, 116 bp; VEGF primer forward, TTA CGG TCT GTG TCC AGT GTA and reverse, TTC TCT GTT ATG TTG CCA GCC, 108 bp.

### Cell proliferation assay

HUVEC and C6 glioma cells (2 × 10^4^) were first incubated in 24-well plates for 24 h. Cells were then treated with 0–180 μg/ml of TMZ and/or 0–180 μg/ml of RG3 for up to 144 h. The same fresh media was replaced every three days. At the end of treatment, 80 μl MTS mix was added into 400 μl of culture media and incubated for 3 h. Optical density (OD) at 592 nm was obtained by Infinite M200 spectrometry (Tecan Inc. Sweden). Cell inhibition rate was calculated as 100 % - (OD of treatment group – OD of background wells) / (OD of control group -OD of background wells) × 100 %. The experiments were performed in triplicates and repeated at least twice. The MTS assay results were validated by cell counting, using hemocytometer after trypan blue staining.

### Cell apoptosis assay

HUVEC and C6 glioma cells (2 × 10^4^) were first seeded in 24-well plates for 24 h. Cells were then treated with 0–180 μg/ml of TMZ and/or 0–180 μg/ml of RG3 for up to 72 h. Treated cells were stained with DAPI. DNA content of DAPI stained cells was analyzed by DNA fluorescent photometer (Nikon P100, Nikon, Tokyo, Japan). Based on DNA content and morphological characteristics, these cells then were classified into three groups: normal cells, apoptotic cells and necrotic cells. A total of 1000 cells of each sample were randomly selected and analyzed. The apoptotic rates were calculated by number of apoptotic cells per total number of cells × 100 %. The experiment was conducted in triplicates and repeated three times.

### Cell cycle assay by flow cytometry (FCM)

Treated cells were fixed in 70 % ethanol and stored at −20 °C. For FCM assay, frozen cell were first washed with PBS and then incubated with propidium iodide (1 mg/ml) at 4 °C for 30 min. Processed cells were analyzed using FACSCanto flow cytometer (BD Biosciences, San Jose, CA) and results were assessed by the Lysis software (BD Biosciences).

### DNA ladder assay

After washing with PBS, treated cells were lysed with the buffer (1 mg/ml proteinase K in 10 mM Tris–HCl, pH 8.0, 150 mM NaCl, 10 mM EDTA and 0.4 % SDS). Cell DNA was then extracted with phenol and chloroform method and resuspended in TE buffer (10 mM Tris HCl, pH 7.5 and 0.2 mM EDTA). The DNA samples were then separated on 2.0 % agarose gel and visualized.

### Real-time PCR

RNA was extracted by RNeasy kit (Qiagen, Shanghai, China) following the manufacturer’s protocol. Real time PCR was performed with SYRB PrimeScript RT-PCR Kit (Takara, Japan) at 95 °C 15 s, 60 °C 1 min and 72 °C for 1 min for 40 cycles. The melting curve was produced to validate the amplification of one target gene. GAPDH was used as the internal reference for the normalization of RNA quantity. VEGF and Bcl-2 mRNA levels in HUVEC were obtained based on threshold cycle (CT) using the formula: the amount of target gene expression =2^-ΔΔCt^, ΔΔCt = Ct of target gene - CT of GAPDH.

### Tumor implantation and treatment

Animal care was handled and maintained in accordance to the established protocols from the Experimental Animal Center of Zhejiang University of Traditional Chinese Medicine. A total of 34 adult male Wistar rats (250 to 300 g) were used for the allograft study. The implantation surgery was performed using the classical published stereotactic protocol [29]. Under general anesthesia with 1 % pentobarbital sodium (i.p.), the rat was placed in a stereotactic frame and fixed with two ear bars. Skin and periosteum were incised and a burr hole was drilled at the coordinates for the right caudate nucleus (1 mm anterior and 3 mm lateral to bregma). Rat C6 glioma cells (5 × 10^6^ in 10 μl serum free media with 1 % agarose) were injected into the caudate nucleus area using a Hamilton microsyringe.

After inoculation for 13 days, 31 out of 34 rats developed tumors based on MRI scanning. These tumor bearing rats were then randomly assigned to five treatment groups (*n* = 6 each, except for the control group *n* = 7). Rats in the maximum tolerated dose of TMZ (MTD TMZ) group were orally administered with 30 mg/kg daily for 3 days. Rats in the low-dose metronomic TMZ (LDM TMZ) group were orally treated with 5 mg/kg/day for eight continuous days. RG3 at 10 mg/kg/day was administrated orally for 8 days to rats in the RG3 group. Rats in the LDM TMZ combined with RG3 group were fed daily with both TMZ (5 mg/kg) and RG3 (10 mg/kg) for 8 continuous days. Rats in the control group were administrated with PBS only. The rat’s weights were checked at twice a week. The tumors were scanned by magnetic resonance imaging (MRI) at the day 13 and 23 after tumor cell injection. All rats were then terminated after the second MRI scanning.

### Magnetic resonance imaging

All rats were subjected to MRI scanning at days 13 and 23 after inoculation of the rat C6 glioma cells. Rats were anesthetized during imaging with 1–1.5 % inhaled isoflurane and monitored during imaging with respiratory monitoring. Images were captured using the Simens3.0 T MRI scanner following a similar procedure described previously [30]. A single-dose pre-load method was applied for perfusion imaging [30]. The contrast agent gadobenate dimeglumine was injected at 0.2 mmol/kg, and repeated with the same dose after 5 min. All the images were transferred to the post-processing workstation for image analysis. Relative cerebral blood volume (rCBV) of each selected layer was calculated based on the pixel analysis and the maximum tumor CBV areas (avoiding normal blood vessels and necrosis areas) were selected.

Tumor necrosis areas were analyzed on T1 + C MRI images using the scanner software. Percentage of necrosis area was calculated by the formula: necrosis area ÷ the tumor section area × 100.

### Immunohistochemical (IHC) staining

IHC staining was performed with rabbit anti-VEGF-A and CD34 antibodies. All primary antibodies were incubated at 4 °C overnight, followed by horseradish peroxidase-conjugated anti-rabbit secondary antibody at room temperature for one hour. Sections were visualized with DAB substrate (Dako). Images were captured at 40X and analyzed by image pro plus 6.0 software.

Positive index of VEGF-A was calculated based upon: the sum of (density of positive area x size of area) divided by total area. For CD34 staining, blood vessel rich “hot-spots” were pictured and microvessel density (MVD) were calculated by image analysis software directly.

### Statistical analysis

Quantitative data with two groups were tested by unpaired Student’s *t*-test using Excel software (Microsoft, Seattle, WA). Quantitative data of over two groups were initially evaluated by analysis of variance followed by Tukey test, to evaluate pairwise comparisons using GraphPad Software (Abacus Concepts, Berkeley, CA). Data transformation by square root was used to meet the normality requirement. *P <* 0.05 was considered as significant.

## Results

### TMZ and RG3 additively inhibited HUVEC proliferation in vitro

**Fig. 1:**
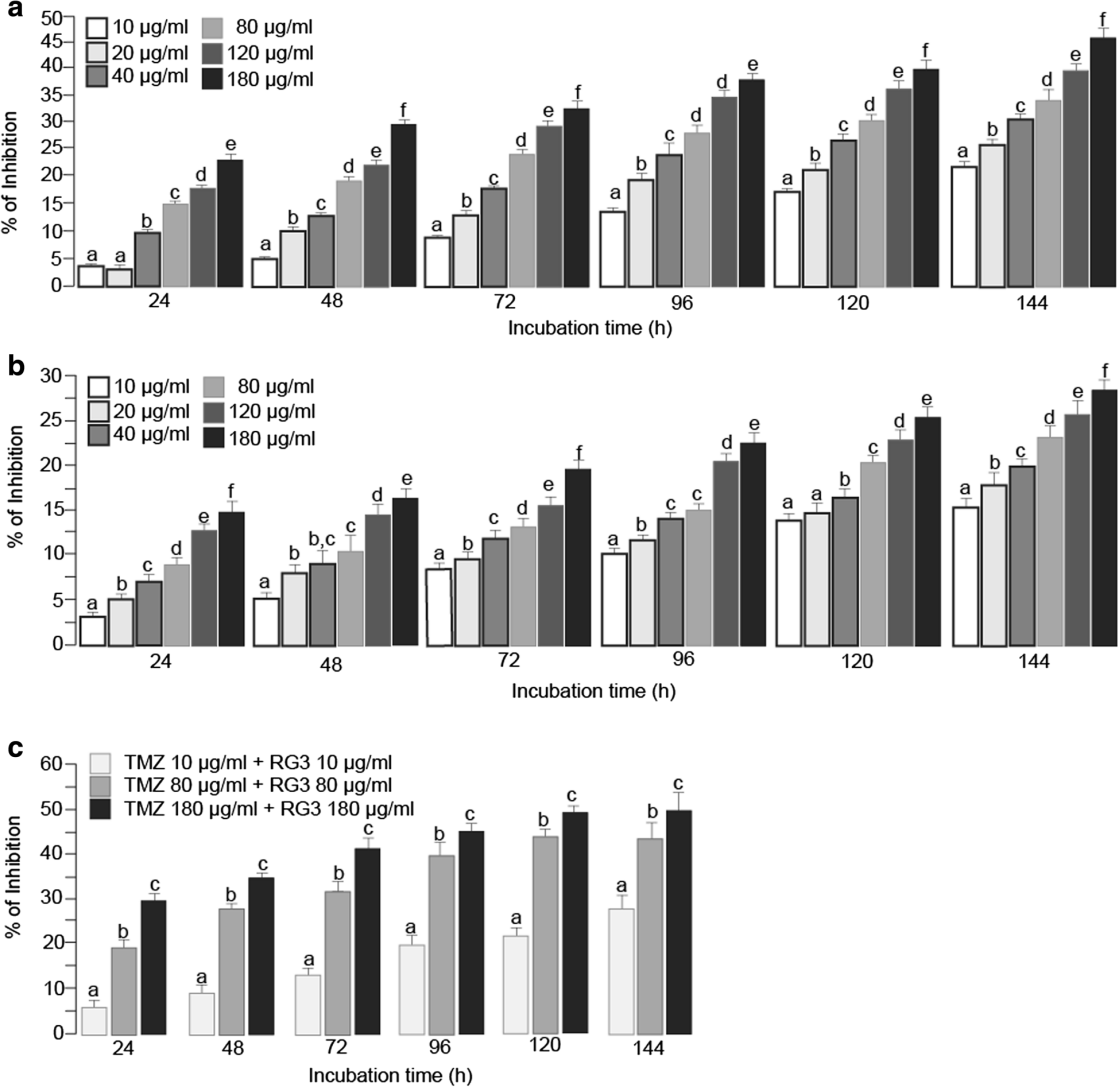
TMZ and RG3 additively inhibited HUVEC proliferation. Cells were seeded in 24 well plates and treated with TMZ and/or RG3 for designated time and doses. **a** TMZ inhibited HUVEC proliferation in a time and dose dependent way. **b** RG3 inhibited HUVEC proliferation in a time and dose dependent way. **c** TMZ and RG3 additively inhibited proliferation of HUVECs. Bars with different letters indicate significant difference (*P* < 0.05) based on post-hoc Tukey analysis

MTS assay showed that TMZ dose and time dependently inhibited HUVEC proliferation. At the concentration of as low as 10 μg/ml and incubation for 24 h, TMZ significantly inhibited HUVEC proliferation by 3.96 ± 0.23 % (*P* < 0.05 compared with the vehicle). As high as 45.73 ± 1.56 % of cell proliferation was inhibited by TMZ at the concentration of 180 μg/ml after 144 h incubation (*P* < 0.0001 compared with the vehicle) (Fig. 1a). RG3 treatment showed similar dose and time dependent inhibition on HUVEC proliferation. RG3 significantly reduced HUVEC proliferation by 3.12 ± 0.66 % at the concentration of 10 μg/ml after 24 h treatment (*P* < 0.05). There was 28.36 ± 1.49% inhibition of cell proliferation by RG3 at 180 μg/ml for 144 h incubation (Fig. 1b).

LMZ and RG3 combined treatment showed additive inhibition on HUVEC proliferation. After incubation with 10 ug/ml TMZ and 10 μg/m RG3 together for 24 h, HUVEC proliferation was reduced by 5.43 ± 0.39 %, whereas only 3.96 ± 0.23 % (*P* < 0.05 compared to combined) and 3.12 ± 0.66 % (*P* < 0.05 compared to combined), respectively, of proliferation inhibition was observed by treatment with TMZ or RG3 alone. Combined treatment with TMZ 180 μg/ml and RG3 180 μg/ml showed 52.37 ± 1.46 % inhibition of HUVEC proliferation after 144 h incubation (Fig. 1c).

### TMZ and RG3 arrested cell cycle and induced apoptosis in HUVECs

**Fig. 2:**
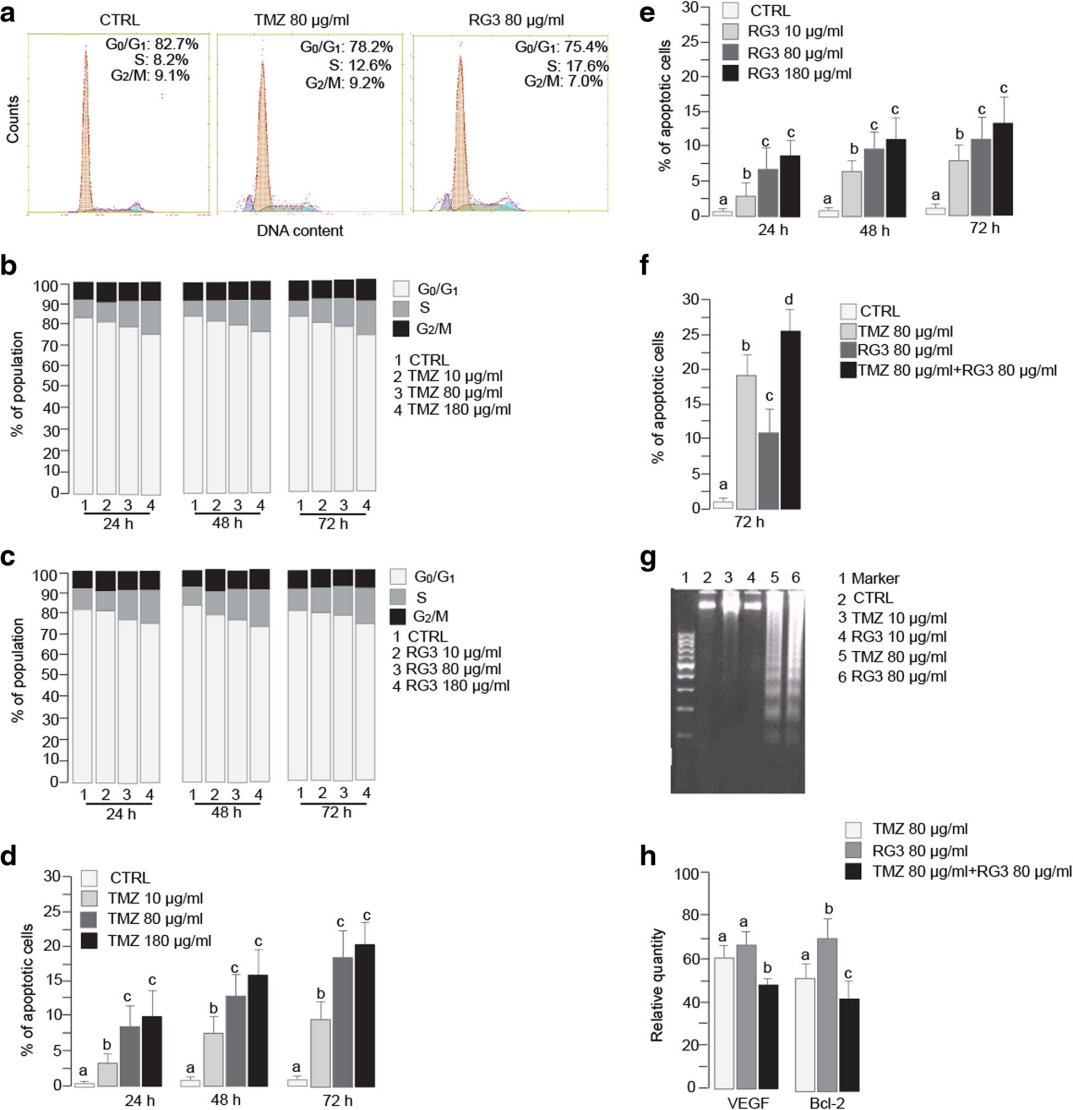
Effect of TMZ and RGs on cell cycle and apoptosis of HUVECs. **a** Representative flow cytometry histograms of cell cycles. Treatment with TMZ (80 μg/ml) or RG3 (80 μg/ml) for 72 h led to accumulation of S phase and appearance of sub-G_0_ apoptotic cells. **b** HUVEC cell cycle arrested at S phase after treatment with TMZ. **c**. HUVEC cell cycle arrested at S phase after treatment with RG3. **d** Rates of apoptotic cells after treatment with TMZ. **e** Rates of apoptotic cells after treatment with RG3. **f** Additive effect of TMZ and RG3 on cell apoptosis. **g** A representative image of DNA ladder after treatment with TMZ (80 μg/ml) or RG3 (80 μg/ml) for 48 h. **h** Realtime PCR data showed that TMZ and RG3 additively reduced both VEGF and Bcl-2 expression compared to vehicle treated control, which was set to 100 %. Bars with different letters indicate significant difference (*P* < 0.05) based on post-hoc Tukey analysis

We then determined the effect of TMZ or RG3 on cell cycles of HUVECs by flow cytometry. The flow cytometry results showed that individual TMZ or RG3 treatment arrested HUVEC cell cycle at S phase, in a dose dependent manner (Fig. 2a, b, c). However, longer time treatment did not significantly increase the effect of cell cycle arrest.

Individual TMZ or RG3 treatment led to dose and time dependent increases in apoptosis of HUVECs (Fig. 2d, e). Combined treatment with TMZ and RG3 showed additive effects on inducing apoptosis ((Fig. 2f). The apoptosis rates of HUVEC treated with individual TMZ (80 μg/ml) or RG3 (80 μg/ml) for 72 h were 18.8 ± 2.7% and 11.4 ± 1.9%, respectively. Combined treatment with TMZ and RG3 additively increased the apoptosis rate to 25.3 ± 3.3% (both *P* < 0.05 vs individual treatment). The apoptosis of HUVEC induced by TMZ or RG3 treatment was validated by characteristic DNA ladder (Fig. 2g).

### TMZ and RG3 reduced VEGF-A and Bcl-2 expression in HUVECs

Real time PCR results showed treatment with either TMZ (80 μg/ml) or RG3 alone (80 μg/ml) for 72 h significantly decreased both VEGF and Bcl-2 mRNA in HUVECs (all *P* < 0.01 compared to control (Fig. 2h). Combined treatment with TMZ and RG3 further decreased mRNA of both VEGF and Bcl-2 in HUVEC (both *P* < 0.05 compared to individual treatment).

### TMZ and RG3 additively suppressed rat C6 glioma proliferation in vitro

**Fig. 3:**
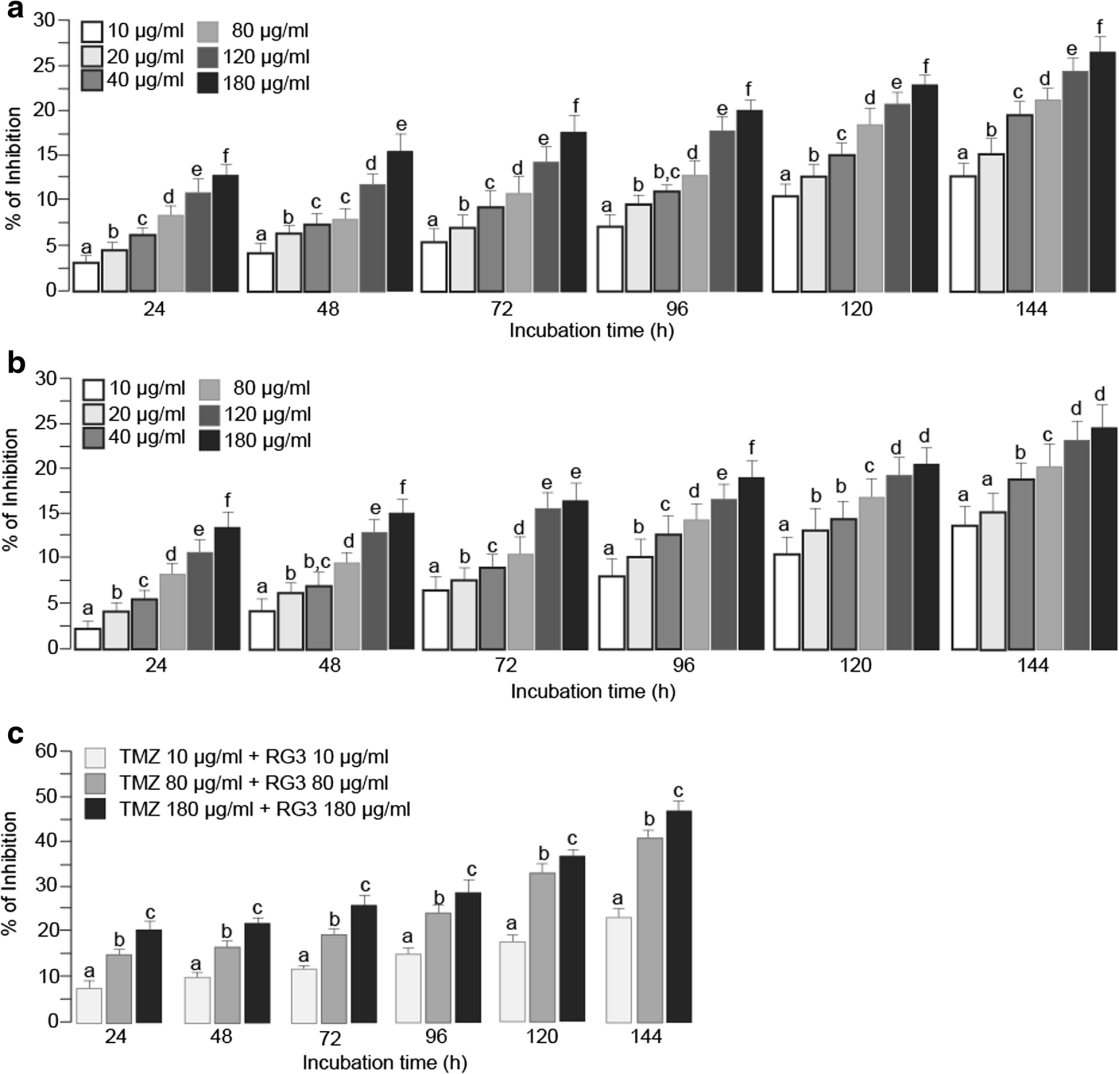
TMZ and RG3 additively inhibited rat C6 glioma cell proliferation. Cells were seeded in 24 well plates and treated with TMZ and/or RG3 for designated time and doses. **a** TMZ inhibited C6 glioma cell proliferation in a time and dose dependent manner. **b** RG3 inhibited C6 glioma cell proliferation in a time and dose dependent way. **c** TMZ and RG3 additively inhibited proliferation of C6 glioma cells. Bars with different letters indicate significant difference (*P* < 0.05) based on post-hoc Tukey analysis

Similar to their inhibitory effects on HUVEC, treatment with individual TMZ and RG3 showed dose and time dependent inhibition on rat C6 glioma cell proliferation (Fig. 3a, b). Combined treatment with RG3 and TMZ showed additive inhibition on C6 proliferation (Fig. 3c).

### The effect of TMZ and RG3 on allografted rat glioma growth

All rats began to decrease body weights 13 days after the inoculation of C6 glioma cells. One rat in the control group died 20 days after inoculation. This allograft experiment was terminated on day 23 after inoculation since three mice in the control group needed to be euthanized due to sickness. Rats in control and MTD LDM groups had the most body weight loss. Treatment with LDM TMZ and RG3 individually or in combination led to similar but less body weight loss (Additional file 1: Table S1).

**Fig. 4:**
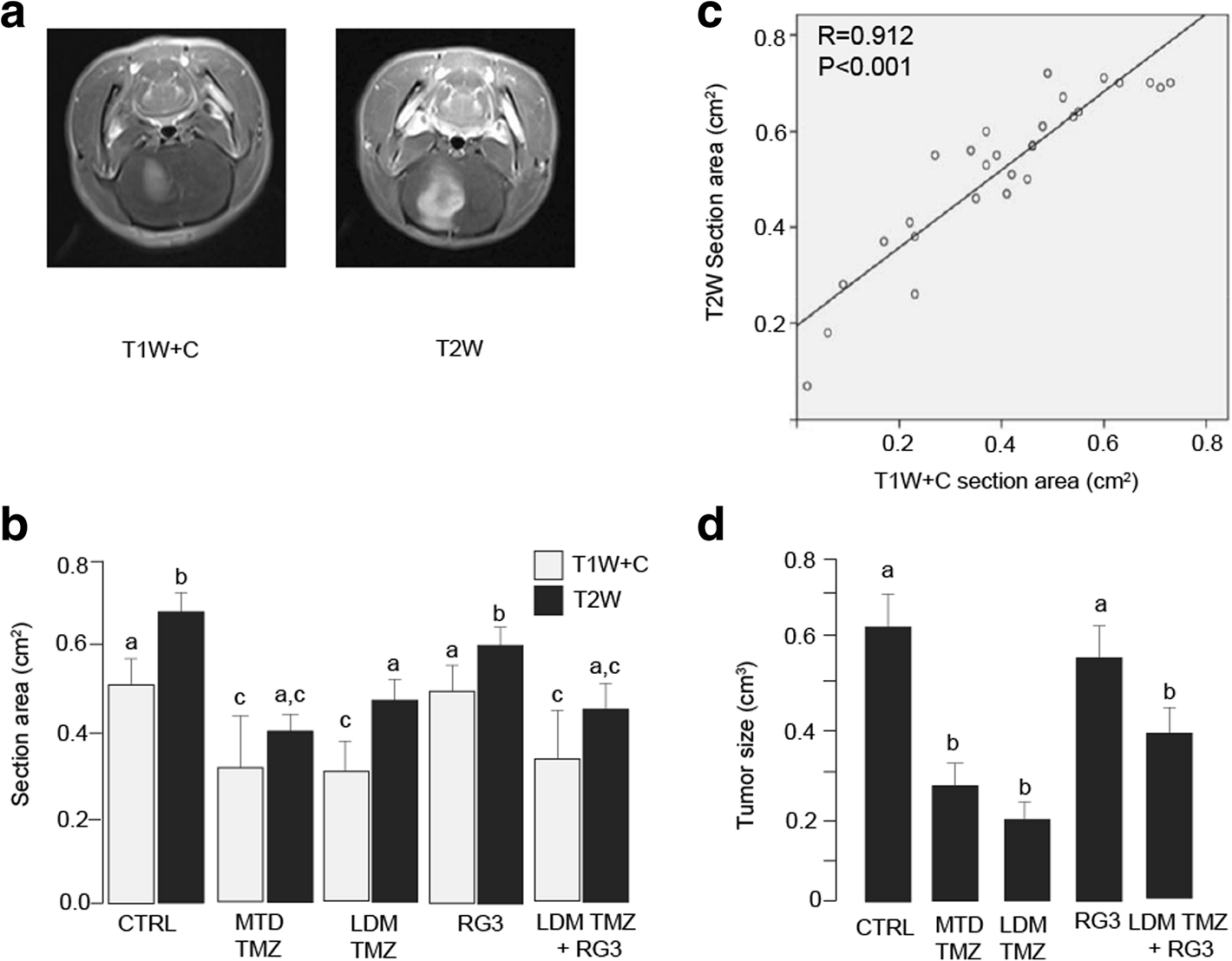
Metronomic TMZ inhibited growth of allografted glioma in rats. **a** Representative T1W + C and T2W MRI images. **b** Section areas of tumors based on T1W + C or T2W MRI images at day 23. **c** A significant correlation was observed between T1W + C and T2W section areas. **d** Sizes of allografted gliomas after different treatments. Bars with different letters indicate significant difference (*P* < 0.05) based on post-hoc Tukey analysis

We then examined the inhibitory effect of treatments on tumor growth based on the T1W + C tumor sectional areas measured on MRI images (Fig. 4a). Our results showed that the sectional area of tumors from control and RG3 groups were 0.51 ± 0.11 and 0.50 ± 0.09 cm^2^ (*P* > 0.05, control vs RG3 group), respectively. The tumor sectional areas from MTD TMZ, LDM TMZ, LDM TMZ and RG3 combined groups were 0.31 ± 0.17, 0.31 ± 0.13 and 0.35 ± 0.15 cm^2^, respectively. Statistical analysis showed that all *P* < 0.05 compared with the control or RG3 group, whereas no significant difference was observed among the above three groups (Fig. 4b). Results of the T2W tumor cross-sectional areas had similar pattern to those in T1W + C (Fig. 4b). The tumor cross-sectional areas for the control and RG3 groups are 0.65 ± 0.07 and 0.59 ± 0.09 cm^2^, respectively (*P* > 0.05, control vs RG3 group). Compared with the above two groups, MTD TMZ, LDM TMZ alone, or LDM TMZ and RG3 combined treatment significantly decreased tumor cross sectional areas to 0.41 ± 0.17, 0.49 ± 0.12 and 0.46 ± 0.14 cm^2^, respectively (all *P* < 0.05 compared to control or RG3 group). There was no significant difference among the above three groups. Linear regression model was applied to determine the correlation between T1W + C and T2W tumor sectional areas. Our results showed a good correlation between these two parameters of tumor cross-sectional areas (R = 0.912, *P* < 0.001) (Fig. 4c).

We also calculated tumor volume based on T1 enhancement from MRI scanning. The average tumor volume of the control and RG3 groups were 0.61 ± 0.17 and 0.53 ± 0.15 cm^3^, respectively (*P* > 0.05). In contrast, MTD TMZ, LDM TMZ alone, LDM TMZ and RG3 combined treatment, all showed a significant decrease in tumor volume, compared to the control or RG3 group (all *P* < 0.05). The mean tumor volumes for MTD, LDM TMZ, and combined LDM TMZ and RG3 groups were 0.29 ± 0.12, 0.19 ± 0.12, and 0.38 ± 0.15 cm^3^, respectively. There was no significant difference in tumor volume among the above three groups.

Our results showed that MTD TMZ, LDM TMZ, and combined treatments had similar effects in induction of necrosis in allografted gliomas. They all significantly increased the necrosis areas in tumors compared to the control or RG3 treatment (Additional file 2: Table S2).

### The effect of TMZ and RG3 on angiogenesis in rat glioma

**Fig. 5:**
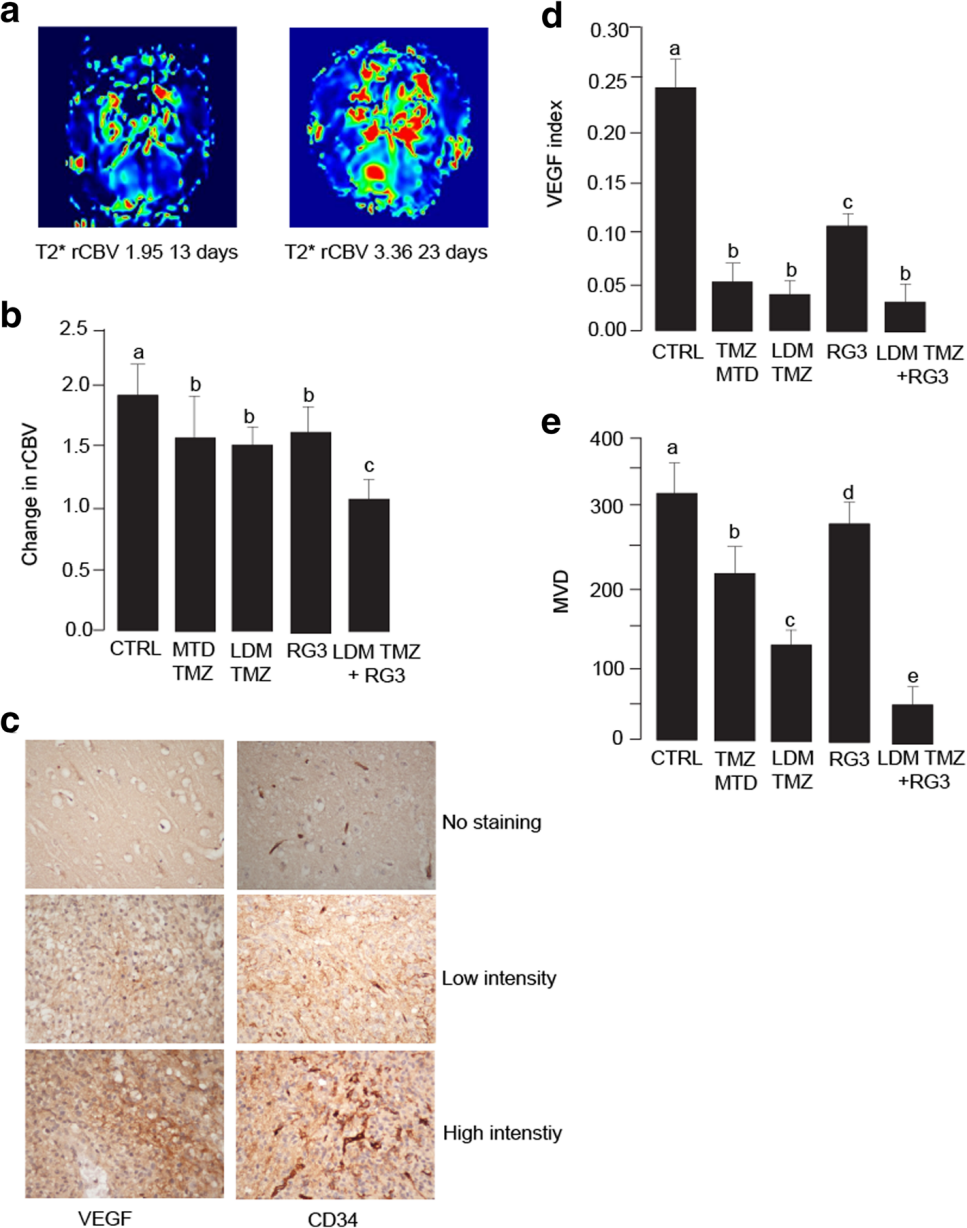
Metronomic TMZ and RG3 additively inhibited angiogenesis in allografted glioma in rats. **a** Representative MRI images showing rCBV of days 13 and 23 after C6 rat glioma cell inoculation. **b** Changes in rCBV from day 13 to 23 after different treatments. **c** Representative images of IHC staining of VEGF and CD34 in allografted gliomas. **d** Quantitative data of VEGF expression in tumors after different treatments. **e** Quantitative data of CD34 expression in tumors after different treatments. Bars with different letters indicate significant difference (*P* < 0.05) based on post-hoc Tukey analysis

The relative cerebral blood volume (rCBV) was measured by dynamic susceptibility-weighted contrast-enhanced perfusion MRI (Fig. 5a). Based on rCBV, tumor blood contents were quantified and compared on the days 13 and 23 after the C6 rat glioma cell inoculation. Compared with day 13, there was 90.5 ± 29.1 % increase in rCBV in the control group on day 23. In contrast, RG3 and MTD TMZ groups showed 65.4 ± 21.5 and 64.9 ± 18.7 %, respectively, increase in rCBV (both *P* < 0.05 compared with the control group, no difference between the two groups). LDM TMZ alone and combined treatment with LDM TMZ and RG3 had even less increase in rCBV. rCBV was increased by 51.2 ± 18.3 % in the LDM TMZ group (*P* < 0.01 compared with the control group, *P* < 0.05 compared to MTD or RG3 group), and by 15.4 ± 12.9 % in combined treatment group (all *P* < 0.01 compared with the control group, or MTD TMZ, or RG3 group, *P* < 0.05 compared with LDM TMZ group) (Fig. 5b).

We then measured the levels of proangiogenic factor VEGF-A by IHC staining on harvested allografted gliomas. Our results showed that VEGF-A expression was very low in the normal rat brain tissues and mainly localized at areas surrounding blood vessels (Fig. 5c*upper left panel*). There were varied levels of VEGF-A expression in glioma cells and tumor endothelial cells after different treatments (Fig. 5c, *middle* and *lower left panel*). Quantitative data showed that RG3 alone had a significant reduction in VEGF expression compared to control. Treatment with MTD TMZ, or LDM TMZ, or combined treatment with LDM TMZ and RG3 further reduced expression of VEGF-A (all *P* < 0.01 compared to the control, *P* < 0.05 compared to RG3 group, but no significant difference among themselves) (Fig. 5d).

Antiangiogenic effect was further evaluated by tumor microvessel density (MVD) using IHC staining of CD34. Normal rat brains had a limited amount of expression in vascular endothelial cells (Fig. 5c, *upper right panel*). Different levels of CD34 staining were localized in glioma vascular endothelial cells (Fig. 5c, *middle* and *lower right panel*). Similar to its effect on VEGF-A expression, RG3 alone significantly decreased MVD in allografted glioma compared to the control group (*P* < 0.05). MTD TMZ had had more potency than RG3 alone in reducing MVD (*P* < 0.05 vs RG3 alone). Treatment with LDM TMZ alone further significantly decreased MVD compared with the RG3 alone or MTD TMZ (both *P* < 0.05). Combined treatment with LDM TMZ and RG3 showed the highest decrease in MVD (*P* < 0.05 compared with all other groups) (Fig. 5e).

## Discussion

In contrast to the standardized schedule, LDM TMZ has been shown to have increased efficacy in the treatment of glioblastoma, with less toxicity. One of the main mechanisms of glioblastoma resistance to TMZ is thought to be mediated by O6-methylguanine-DNA methyltransferase (MGMT). It has been speculated that LDM TMZ will deplete MGMT and accumulate higher levels of O6-mehtylated DNA adducts, thus reducing the chemotherapeutic resistance [31]. Furthermore, recent data imply that antiangiogeneis is the main mechanism attributing to this enhanced efficacy in treatment of glioblastoma. In vitro studies reveal that LDM TMZ treatment significantly inhibits endothelial cell proliferation, and endothelial cells are more sensitive to TMZ than glioblastoma cells [32, 33]. The LDM TMZ demonstrated the antiangiogenic effect in a mouse model of orthotopic xenografted glioma [13]. Our current in vivo data show that LDM TMZ is even more potent than the MTD on angiogenesis inhibition in glioma. In addition, our in vitro and in vivo data demonstrate that LDM TMZ treatment reduces the levels of proangiogenetic factor VEGF. In consistence with our result, LDM TMZ has been shown to prolong glioblastoma patient progression-free interval and overall survival with less toxicity in several clinical trials [14–17]. However, LDM TMZ alone still has a limited effect on glioblastoma relapse. It is of clinical significance to identify antiangiogenesis agents to improve its efficacy in the treatment of glioblastoma.

RG3 is the active component of ginseng, which is a popular herbal medicine used to proactively promote health, vitality, and longevity. In contrast to other regular chemotherapeutic drugs, RG3 has been shown to be relatively safe and effective, with little side effects. Its antiangiogenesis effects have been demonstrated in multiple tumor models, along with its antitumor effects [34–38]. In addition, chronic use of RG3 inhibits glioma growth via Akt dependent pathway [28] and inhibits glioma cell proliferation by changing redox status [39]. Our current study shows that RG3 alone significantly inhibits proliferation, arrests the cell cycle and induces apoptosis in HUVEC through reducing VEGF and Bcl-2 expression. RG3 demonstrates a modest antitumor and antiangiogenesis effect in allografted gliomas. All these data suggest that RG3 is an excellent candidate antiangiogenesis drug to be used in combination therapy.

The novel finding of this study is that combined treatment with LDM TMZ and RG3 additively inhibits glioma cell growth in vitro, and suppresses angiogenesis both in vitro and in vivo. It is noted that this additive antiangiogenesis effect is observed in orthotopic allografted glioma in immune competent rats. Their additive antiangiogenesis effect is through arresting the cell cycle, inducing apoptosis and reducing expression of VEGF and Bcl-2 in blood vessel endothelial cells. Though the mechanism attributing to this additive effect is still unknown, RG3 has been shown to increase the concentration of the chemotherapeutic drug paclitaxel in tumors [40]. RG3 combined with gemcitabine inhibits angiogenesis of lung cancer and improves survival of tumor-bearing mice [41]. Combined use of TMZ with other drugs has shown their improved effects compared with TMZ alone [42, 43]. Based on body weight change in glioma bearing rats, combination therapy with LDM TMZ and RG3 have less toxicity to the host than MTD TMZ. To the best of our knowledge, this study is the first to report the additive antiangiogenesis effect of combined therapy with LDM TMZ and RG3 in the treatment of gliomas.

Antiangiogenesis therapy has recently been added to the panel of cancer therapeutics. Predictive biomarkers are important to evaluate of efficacy of treatment. MVD is closely related to tumor growth, metastasis and prognosis. Due to tumor heterogeneity, invasive biopsy or partial biopsy alone may not fully reflect the entire tumor growth. In addition, biopsy has the limitations of its use in glioblastoma particularly because of the ethical issue to obtain tissue specimens from brain tumors. Serological markers such as VEGF and bFGF have a good correlation with glioblastoma blood vessel density, but are vulnerable to a variety of metabolic factors that may interfere in vivo. Conventional CT and MRI inspection, based on morphological changes and other indirect signs, are difficult to assess tumor angiogenesis. CT perfusion imaging has a good correlation with tumor angiogenesis [44], but has radiation hazards. Dynamic susceptibility-weighted contrast-enhanced perfusion MRI is the most commonly used non-invasive method to assess cerebral perfusion and tumor vascularity in the central nervous system [30, 45–48]. Our data show that rCBV measured by this type of MRI has a pattern similar to the angiogenesis biomarkers VEGF and MVD. This non-penetrative measurement might be an objective biomarker in the assessment of antiangiogenesis therapies [49].

## Conclusions

This study provides supporting data showing that LDM TMZ has improved efficacy and decreased side effects. Notably, our data provide strong preclinical evidence that combined use of LDM TMZ and RG3 have additive antiangiogenic effects. The combined additive effect suggests that RG3 has the potential to further increase the efficacy of LDM TMZ in the treatment of glioblastoma.

## Additional files

Additional file 1: Table S1. Body weight changes in rats after treatments. (DOC 32 kb) Additional file 2: Table S2. Comparison of tumor necrosis areas among treatment groups. (DOC 32 kb)

## Abbreviations

FCM: Flow cytometry
HUVEC: Human umbilical vein endothelial cells
LDM: Low dose metronomic
MRI: Magnetic resonance imaging
MTD: Maximum tolerated dose
OD: Optical density
rCBV: Relative cerebral blood volume
TMZ: Temozolomide
