# Supplementary material for: Additive antiangiogenesis effect of ginsenoside Rg3 with low-dose metronomic temozolomide on rat glioma cells both in vivo and in vitro
Source: J Exp Clin Cancer Res. 2016 Feb 13;35:32. doi: 10.1186/s13046-015-0274-y (PMC4752767; doi:10.1186/s13046-015-0274-y)
Supplement: Additional file 1: Table S1. — Body weight changes in rats after treatments. (DOC 32 kb) [file 13046_2015_274_MOESM1_ESM.doc]

Additional file 1: Table S1. Body weight changes in rats after treatments.

| Groups | n | Weight change (g)* |
| --- | --- | --- |
| Control | 6 | -43.56 ± 32.2^a^ |
| MTD TMZ | 6 | -37.12 ± 20.05^a^ |
| LDM TMZ | 6 | -7.28 ± 18.81^b^ |
| Rg3 | 6 | -6.67 ± 12.39^b^ |
| LDM TMZ+Rg3 | 6 | -16.85 ± 17.46^b^ |

* Number with different label indicates significant difference (P<0.05).
