# Supplementary material for: Additive antiangiogenesis effect of ginsenoside Rg3 with low-dose metronomic temozolomide on rat glioma cells both in vivo and in vitro
Source: J Exp Clin Cancer Res. 2016 Feb 13;35:32. doi: 10.1186/s13046-015-0274-y (PMC4752767; doi:10.1186/s13046-015-0274-y)
Supplement: Additional file 2: Table S2. — Comparison of tumor necrosis areas among treatment groups. (DOC 32 kb) [file 13046_2015_274_MOESM2_ESM.doc]

Additional file 2: Table S2. Comparison of tumor necrosis areas among treatment groups

| Groups | n | Number (%) | Necrosis areas (%)* |
| --- | --- | --- | --- |
| Model | 6 | 1 (33.3) | 0.05 ±0.07^a^ |
| MTD TMZ | 6 | 4 (66.7) | 0.12±0.07^b^ |
| LDM TMZ | 6 | 5 (83.7 ) | 0.14±0.11^b^ |
| Rg3 | 6 | 3 (50.0 ) | 0.06±0.21^a^ |
| LDM TMZ+Rg3 | 6 | 5 (83.7) | 0.18±0.05^b^ |

* Number with different label indicates significant difference (P<0.05).
